# Gαi3 nuclear translocation causes irradiation resistance in human glioma cells

**DOI:** 10.18632/oncotarget.17043

**Published:** 2017-04-11

**Authors:** Shang Cai, Ya Li, Jin-Yu Bai, Zhi-Qing Zhang, Yin Wang, Yin-Biao Qiao, Xiao-Zhong Zhou, Bo Yang, Ye Tian, Cong Cao

**Affiliations:** ^1^ Department of Radiotherapy and Oncology, The Second Affiliated Hospital of Soochow University, Suzhou, China; ^2^ Institute of Neuroscience, Soochow University, Suzhou, China; ^3^ Department of Orthopedics, The Second Affiliated Hospital of Soochow University, Suzhou, China; ^4^ Department of Surgery, The Third Hospital affiliated to Soochow University, Suzhou, China

**Keywords:** glioma, irradiation, Gαi3, DNA-PKcs

## Abstract

We have previously shown that Gαi3 is elevated in human glioma, mediating Akt activation and cancer cell proliferation. Here, we imply that Gαi3 could also be important for irradiation resistance. In A172 human glioma cells, Gαi3 knockdown (by targeted shRNAs) or dominant-negative mutation significantly potentiated irradiation-induced cell apoptosis. Reversely, forced over-expression of wild-type or constitutively-active Gαi3 inhibited irradiation-induced A172 cell apoptosis. Irradiation in A172 cells induced Gαi3 translocation to cell nuclei and association with local protein DNA-dependent protein kinase (DNA-PK) catalytic subunit. This association was important for DNA damage repair. Gαi3 knockdown, depletion (using Gαi3 knockout MEFs) or dominant-negative mutation potentiated irradiation-induced DNA damages. On the other hand, expression of the constitutively-active Gαi3 in A172 cells inhibited DNA damage by irradiation. Together, these results indicate a novel function of Gαi3 in irradiation-resistance in human glioma cells.

## INTRODUCTION

Glioma is the most common primary central nervous system (CNS) malignancy. It is a major health threat [1–3]. Each year, glioma will cause significant cancer-related death [1–3]. Postoperative irradiation and temozolomide (TMZ) chemotherapy are the standard clinical treatments for glioma [4–6]. Yet, the overall survival has not been significantly improved over the past decades [4–6]. The prognosis of high-grade glioma, including glioblastoma, has been poor [1, 6, 7]. One possible cause is the overwhelming resistance to current irradiation (and chemotherapy) [1, 6, 7].

G protein α inhibitory subunit (Gαi) couples with GPCRs (G-protein coupled receptors) [8] to inhibit adenylate cyclase (AC) [8]. Recently, our group [9–11] and others [12] have discovered an un-anticipated function of Gαi: transducing Akt-mTOR signaling by receptor tyrosine kinases (RTKs). We have previously found that Gαi protein was required for EGFR (epidermal growth factor receptor)- and FGFR (fibroblast growth factor receptor)-induced activation of Akt signaling [9–11]. In our model, Gαi could couple with EGFR/FGFR to activate the adaptor protein (*i.e*. Gab1), which mediates activation of downstream Akt signaling [9–11].

There are at least three Gαi subunits, including Gαi1, Gαi2 and Gαi3 [8]. Our recent study has shown that Gαi3 is over-expressed in human glioma cells, which is required for Akt activation and cancer cell proliferation [11]. The results of the current study indicate that Gαi protein could also be important for irradiation resistance.

## RESULTS

### Silencing Gαi3 sensitizes irradiation-induced glioma cell death

**Figure 1:**
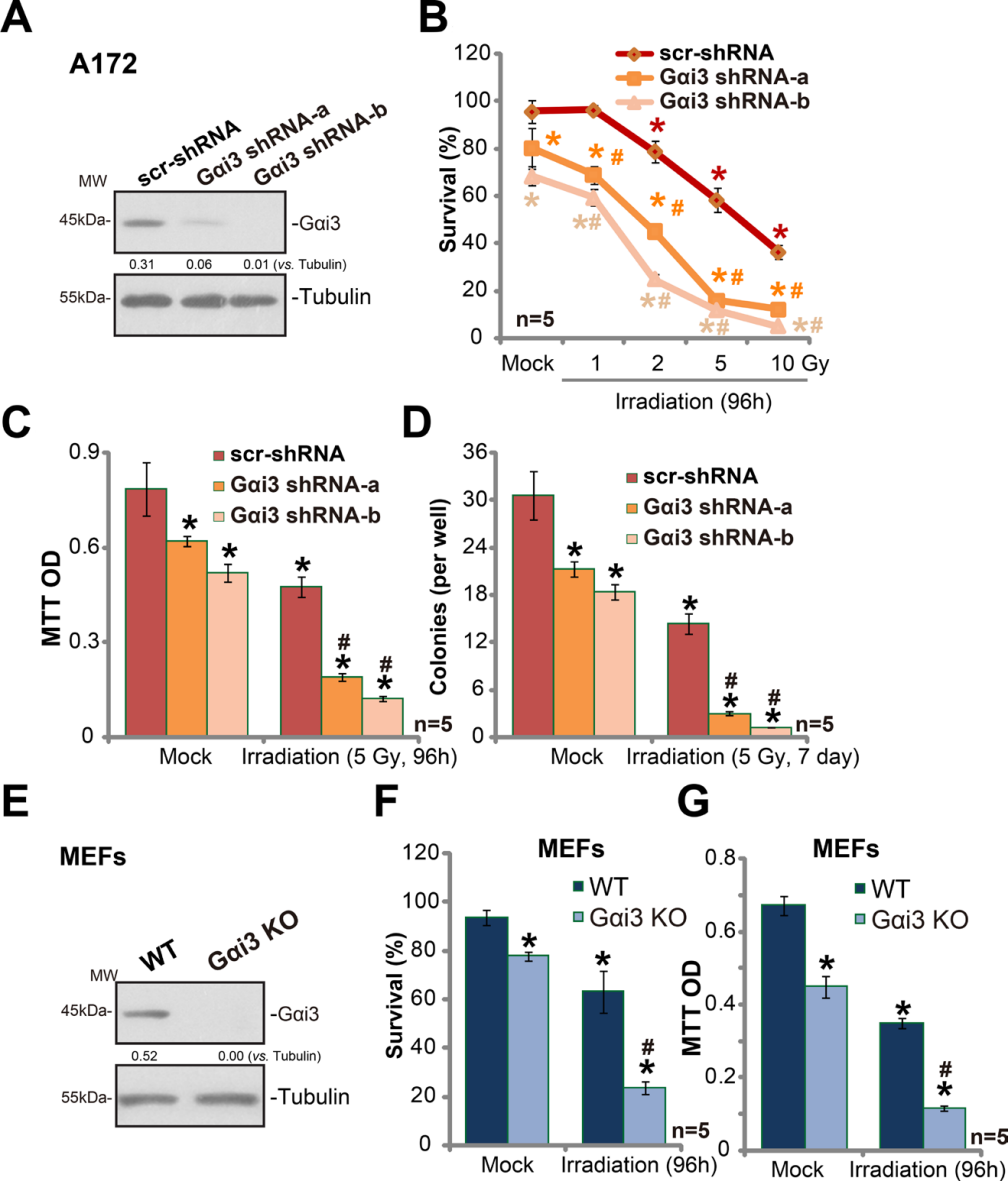
Silencing Gαi3 sensitizes irradiation-induced glioma cell death Western blotting tested expression of listed proteins in stable A172 cells with Gαi3 shRNA (“−a/−b”) or scramble control shRNA (“scr-shRNA”) (**A**); A172 cells were also subjected to irradiation (at indicated intensity) and cultured for indicated time, listed assays were performed to test cell survival/death (**B**–**D**). Expression of listed proteins in wild-type (WT) and Gαi3 knockout (KO) MEFs was shown (**E**); MEFs were irradiated (5 Gy) and cultured for additional 96 hours. Afterwards, MEFs were subjected to trypan blue staining assay (**F**) and MTT assay (**G**). For all the assays, the exact same number of viable cells of different genetic background was initially plated into each well (Same for all Figures). Same set of lysate samples were run in sister gels (A and E). “Mock” stands for un-irradiated cells (Same for all Figures). “*n* = 5” means five replicate wells (Same for all Figures). Bars stand for mean ± SD (Same for all Figures). *p < 0.05 *vs*. “Mock” of “scr-shRNA” A172 cells or WT MEFs. ^#^*p* < 0.05 *vs*. “Irradiation” of “scr-shRNA” A172 cells (B–D) or WT MEFs (F and G). Experiments in this figure were repeated three times, with similar results obtained.

In order to study the potential function of Gαi3 in irradiation resistance, shRNA strategy was applied. As described previously [11], two distant lentiviral shRNAs against non-overlapping sequence of Gαi3 were utilized. The two were named as “Gαi3 shRNA-a” and “Gαi3 shRNA-b”. As shown in Figure 1A, the two Gαi3 shRNAs silenced Gαi3 in human glioma A172 cells. These Gαi3-silenced A172 cells and control cells were treated with various degree (0–10 Gy) of irradiation. Trypan blue staining assay results in Figure 1B demonstrated that A172 cells with Gαi3 shRNA were significantly more sensitive to irradiation than the control A172 cells. Irradiation led to significantly more A172 cell death after Gαi3 knockdown (Figure 1B). The IC-50 of irradiation, or the intensity that kills 50% of A172 cells, decreased from over 6 Gy to less than 1.5 Gy after Gαi3 silence (Figure 1B). MTT assay results (Figure 1C) and colony formation assay (Figure 1D) further confirmed that Gαi3 knockdown significantly facilitated irradiation (5 Gy)-induced killing of A172 cells. Notably, Gαi3 shRNA-b was more efficient in silencing Gαi3 (than Gαi3 shRNA-a, Figure 1A), it was also more dramatic in sensitizing irradiation-induced A172 cell death (Figure 1B–1D). Notably, Gαi3 silence alone also induced minor/moderate A172 cell death (Figure 1B–1D), which was also reported early [11].

The results above suggested that Gαi3 might be important in irradiation resistance. To further support this hypothesis, Gαi3 knockout (“KO”) mouse embryonic fibroblasts (MEFs) [11] were utilized.. Trypan blue assay results in Figure 1F and MTT assay results in Figure 1G confirmed that Gαi3 KO MEFs were significantly more vulnerable to irradiation (5 Gy) than the wild-type (“WT”) MEFs. For instance, 96 hours after irradiation (5 Gy), 63.3 ± 8.6 % of WT MEFs were still alive, yet only 23.6 ± 2.6% of Gαi3 KO MEFs were trypan blue negative (Figure 1F). Together, these results demonstrate that Gαi3 silence or depletion could lead to irradiation-sensitization in glioma cells.

### Silencing Gαi3 sensitizes irradiation-induced glioma cell apoptosis

**Figure 2:**
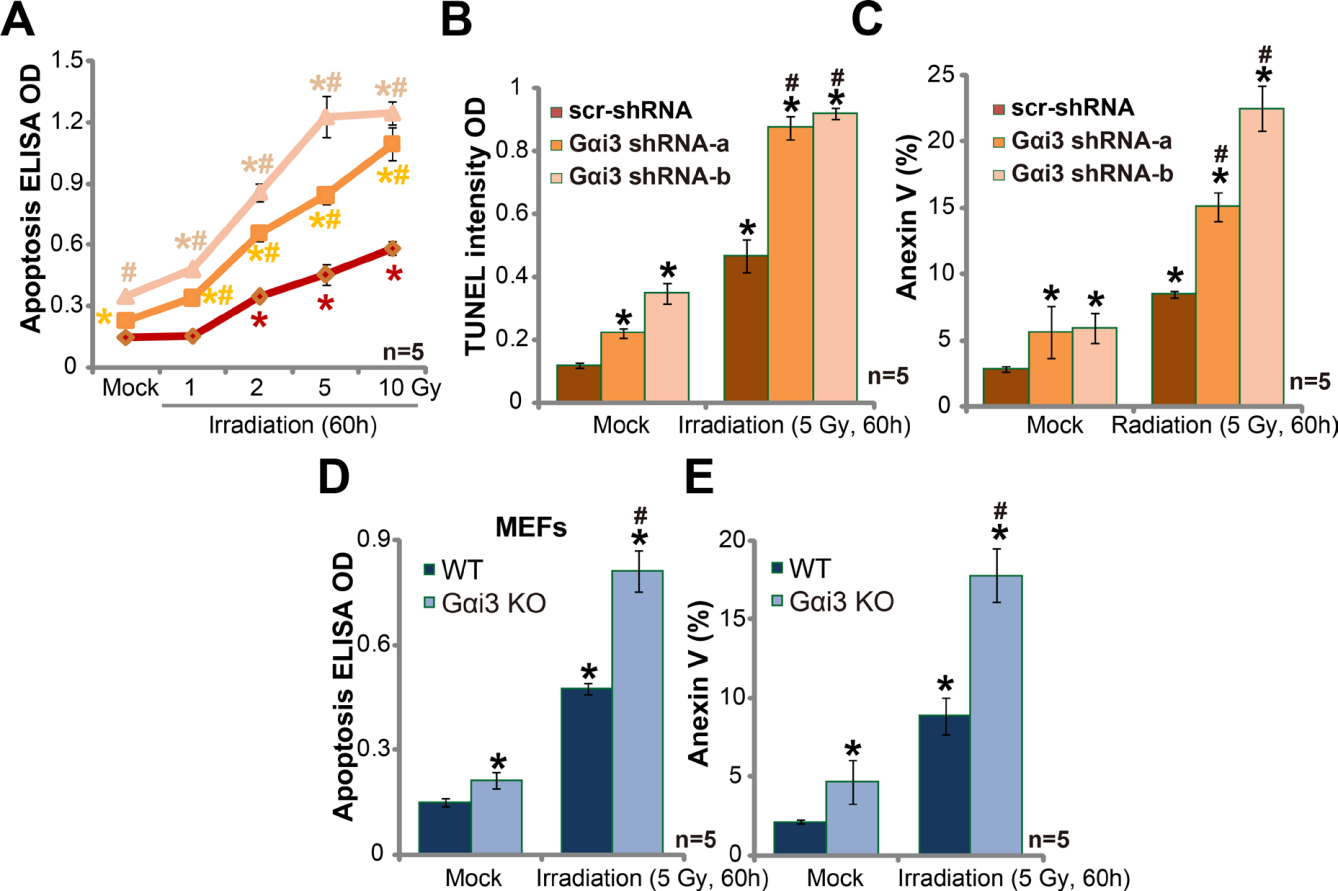
Silencing Gαi3 sensitizes irradiation-induced glioma cell apoptosis A172 cells with Gαi3 shRNA (“−a/−b”) or scramble control shRNA (“scr-shRNA”) (**A**–**C**), as well as wild-type (WT) and Gαi3 knockout (KO) MEFs (**D**–**E**) were treated with irradiation (at indicated intensity) and cultured for additional 60 hours; Afterwards, cell apoptosis was tested by the listed assays. *p < 0.05 *vs*. “Mock” of “scr-shRNA” A172 cells (A-C) or WT MEFs (D–E). ^#^*p* < 0.05 *vs*. “Irradiation” of “scr-shRNA” A172 cells (A–C) or WT MEFs (D–E).

It is known that irradiation kills cancer cells via inducing cell apoptosis [13, 14]. We next wanted to know the potential effect of Gαi3 in the process. In line with our previous studies [10, 11, 15, 16, 17], various apoptosis assays were applied, including Histone DNA apoptosis ELISA assay, TUNEL intensity assay and Annexin V staining assay. As expected, irradiation treatment in A172 cells induced significant apoptosis, which was evidenced by increase of Histone DNA apoptosis ELISA OD (Figure 2A), TUNEL intensity OD (Figure 2B) and percentage of Annexin V positive cells (Figure 2C). Remarkably, Gαi3 silence by targeted shRNA dramatically facilitated irradiation-induced A172 cell apoptosis (Figure 2A–2C). Gαi3 shRNA alone (no irradiation) also induced minor A172 cell apoptosis (Figure 2A–2C). Gαi3 KO MEFs were again utilized. As demonstrated, irradiation (5 Gy) induced significantly more apoptosis in Gαi3 KO MEFs (as compared to WT MEFs, Figure 2D and 2E). For instance, after irritation, 17.6 ± 1.5% of Gαi3 KO MEFs were apoptotic (Annexin V positive), compared to only 6.3 ± 1.9% in WT MEFs (Figure 2E). Basal apoptosis activation was slightly higher in Gαi3 KO MEFs than in the WT MEFs (Figure 2D and 2E) [11].

### Exogenous Gαi3 over-expression in A172 cells cause irradiation resistance

**Figure 3:**
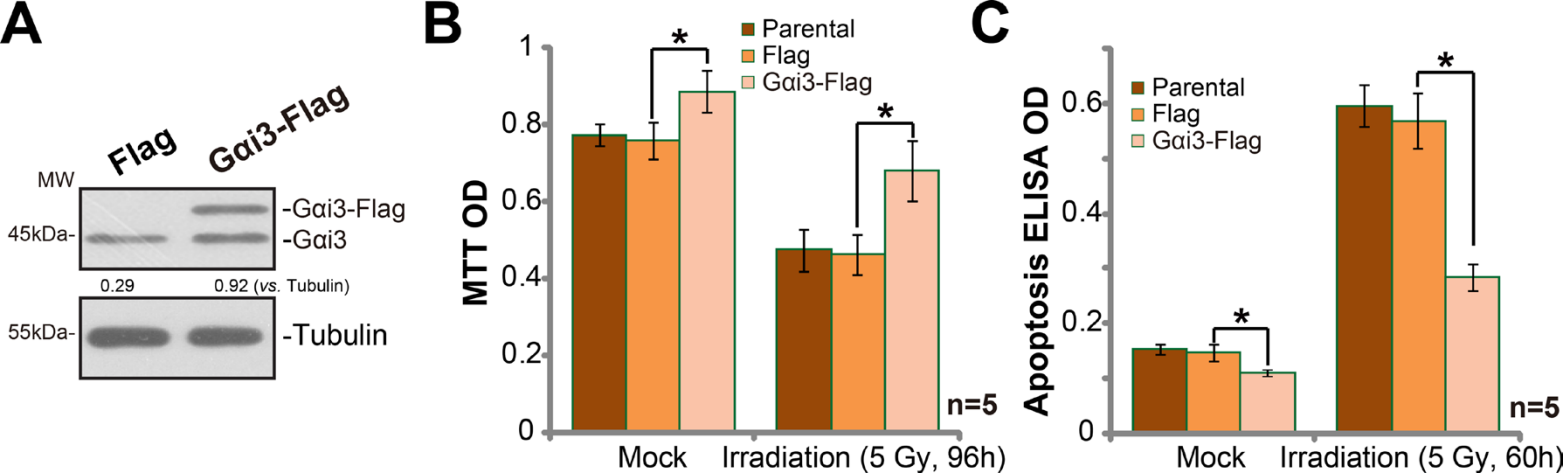
Exogenous Gαi3 over-expression in A172 cells cause irradiation resistance (**A**) Western blotting analysis results showed expression of Gαi3 (endogenous and exogenous) in stable A172 cells with the Flag-tagged Gαi3 or empty vector (pSuper-puro-Flag, “Flag”). Cells were treated with irradiation (5 Gy) and cultured for indicated time; Cell death (MTT OD reduction, (**B**)) and apoptosis (Histone DNA ELISA assay, (**C**)) were tested. “Parental” stands for control parental A172 cells. *p < 0.05.

Based on the results above, we would speculate that Gαi3 over-expression shall cause irradiation resistance. Thus, wild-type (“WT”) Gαi3 construct (see our previous study [10, 11]) was introduced to A172 cells. Via puromycin selection, the stable cells with the construct were established. Western blotting assay results in Figure 3A confirmed the expression of exogenous Gαi3 (Flag-tagged) in the stable cells. Significantly, irradiation-induced A172 cell death (MTT OD reduction, Figure 3B) and apoptosis (Histone DNA ELISA OD increase, Figure 3C) were dramatically inhibited in Gαi3-over-expressed A172 cells. Thus, Gαi3 over-expression led to irradiation resistance in glioma cells.

### Irradiation sensitivity is altered with Gαi3 mutation in A172 cells

**Figure 4:**
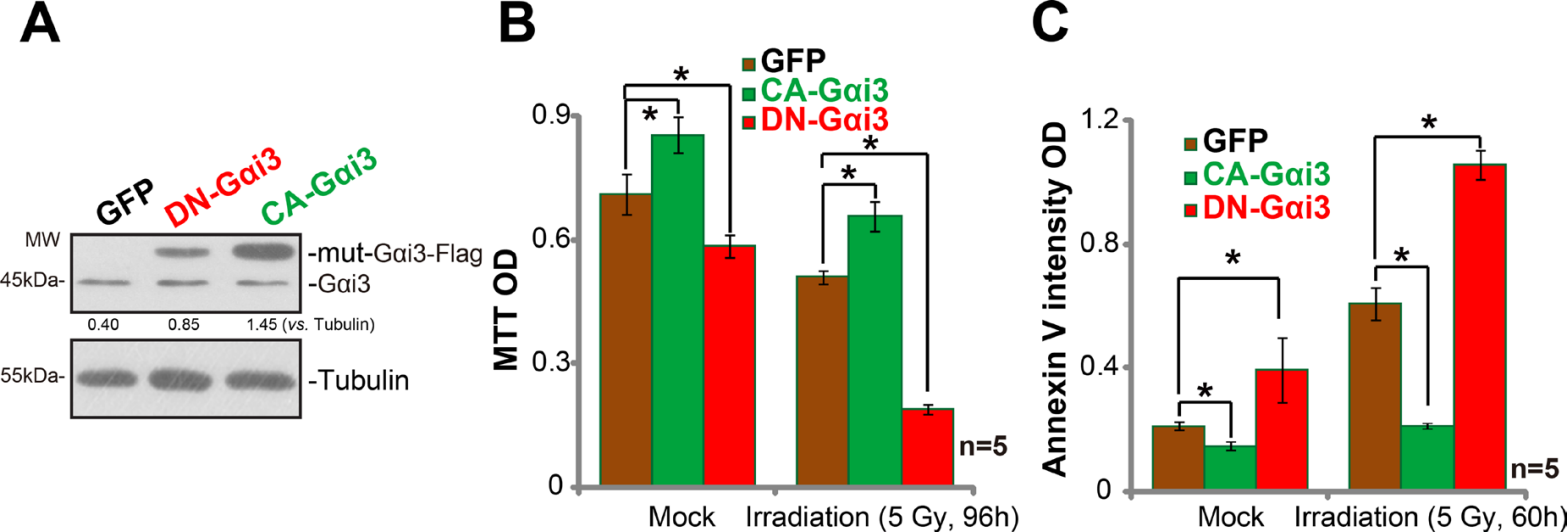
Irradiation sensitivity is altered with Gαi3 mutation in A172 cells Western blotting assay results showed expression of Gαi3 (endogenous and mutant) in stable A172 cells with the dominant-negative Gαi3 (G202T, “DN-Gαi3”), the constitutively-active Gαi3 (Q204L, “CA-Gαi3”) or the empty vector (pGCL-GFP-puro, “GFP”) (**A**). Cells were also treated with irradiation (5 Gy) and cultured for indicated time; Cell death (MTT OD reduction, (**B**)) and apoptosis (Histone DNA ELISA assay, (**C**)) were tested. *p < 0.05.

Next, mutation strategies were employed to potentially alter the activity of Gαi3 in A172 cells. As discussed in our previous studies [9, 11], the dominant-negative Gαi3 (DN-Gαi3), which has a conserved Gly (G) residue replaced by Thr (T) in the G3 box [9, 10, 11], was introduced to A172 cells (Figure 4A). The DN-Gαi3 shall compete with the wt-Gαi3 for binding with other proteins [18, 19]. Significantly, irradiation-induced A172 cell death (Figure 4B) and apoptosis (Figure 4C) were remarkably potentiated with the Gαi3 DN mutation. On the other hand, a constitutively-active Gαi3 (Q204L, CA-Gαi3) [9] was transfected to A172 cells, and stable cells were again established. Results in Figure 4A confirmed CA-Gαi3 (Flag-tagged) expression in the stable A172 cells (Figure 4A). Remarkably, A172 cells with CA-Gαi3 were protected from irradiation (Figure 4B and 4C). Irradiation-induced A172 cell death (Figure 4B) and apoptosis (Figure 4C) were largely inhibited after CA-Gαi3 expression. These results together indicate that change of Gαi3 activity could alter irradiation sensitivity in A172 cells.

### Irradiation induces Gαi3 nuclear translocation and association with DNA-PKcs

**Figure 5:**
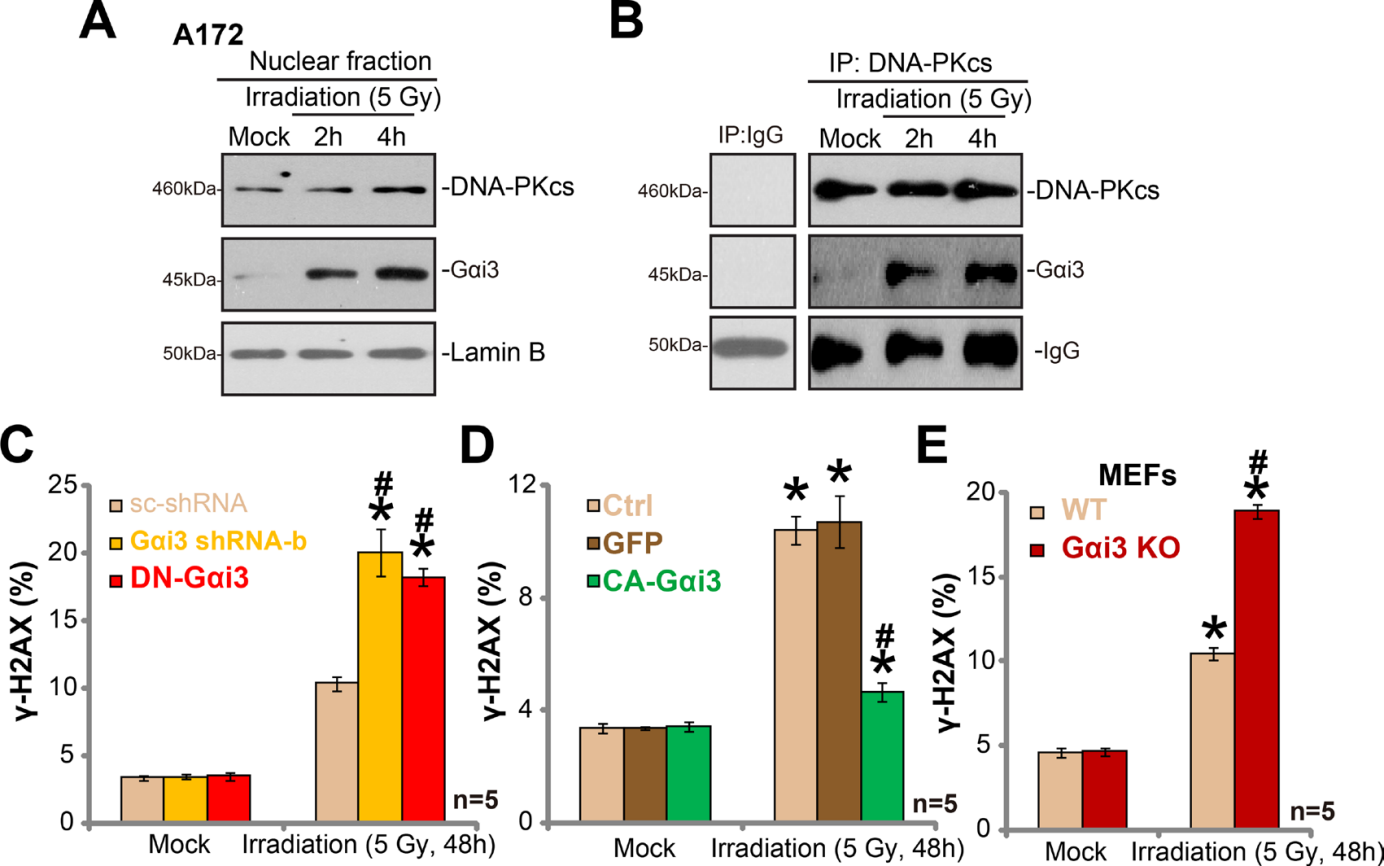
Irradiation induces Gαi3 nuclear translocation and association with DNA-PKcs A172 cells were treated with irradiation (5 Gy) for 2 and 4 hours, nuclear fractions were isolated, expression of listed protein was tested (**A**), Lamin-B is the nuclear marker protein). The association between DNA-PKcs and Gαi3 in cell nuclei was tested by Co-IP assay (**B**). Stable A172 cells with scramble control shRNA (“scr-shRNA”), Gαi3 shRNA (“-b”), the dominant-negative Gαi3 (G202T, DN-Gαi3) or the constitutively-active Gαi3 (Q204L, CA-Gαi3), as well as wild-type (WT) and Gαi3 knockout (KO) MEFs, were treated with irradiation (5 Gy) and cultured for 48 hours, DNA damage was tested by γ-H2AX FACS assay, and γ-H2AX-postive cell ratio was recorded (**C**–**E**). *p < 0.05 *vs*. “Mock”. # *p* < 0.05 *vs*. “Irradiation” of “scr-shRNA” A172 cells (C), “GFP” A172 cells (D) or WT MEFs (E).

It is known that irradiation induces DNA damages, which leads to subsequent cell apoptosis [20–22]. DNA repair mechanisms could however repair damaged DNA, causing irradiation resistance [20–22]. One of major protein complex for DNA repair is DNA-dependent protein kinase (DNA-PK). DNA-PK is primarily composed of the 460-kDa catalytic subunit (DNA-PKcs) and the Ku hetero-dimer (Ku-70 and Ku-80) [23, 24]. Intriguingly, we showed that irradiation treatment in A172 cells induced Gαi3 translocation to nuclei (Figure 5A). Basal Gαi3 level in nuclei, as expected, was few (Figure 5A). Following the irradiation, the Gαi3 level in the cell nuclei was significantly increased (Figure 5A), indicating nuclear translocation. Remarkably, the co-immunoprecipitation assay results showed that nuclei-translocated Gαi3 formed a complex with local protein DNA-PKcs (Figure 5B). Considering that DNA-PKcs is critical for DNA damage repair [20–22], we proposed that Gαi3 could also be important for DNA repair. Indeed, we found that irradiation-induced DNA-damage, tested by γ-H2AX increase [25–27], was significantly potentiated with Gαi3 silence (by “Gαi3 shRNA-b”) or DN mutation in A172 cells (Figure 5C). Reversely, expression of CA-Gαi3 inhibited DNA damages by irradiation (Figure 5D). Further, as compared to the WT MEFs, an increase of γ-H2AX staining (indicating DNA damage) was noticed in irradiated Gαi3 KO MEFs (Figure 5E). Notably, basal DNA-damage or γ-H2AX staining was unchanged by above Gαi3 genetic modifications (Figure 5C–5E). Together, our results imply that irradiation induces Gαi3 nuclear translocation and association with DNA-PKcs, which apparently is crucial for DNA-damage repair and irradiation resistance.

## DISCUSSION

The results of this study suggest that Gαi3 could be a key resistance factor of irradiation in glioma cells. Gαi3 depletion significantly potentiated irradiation-induced cell apoptosis. On the other hand, forced over-expression of Gαi3 inhibited irradiation-induced A172 cell apoptosis. Meanwhile, irradiation sensitivity in A172 cells was potentiated when expressing DN-Gαi3, but was reduced after CA-Gαi3 expression. Mechanistic study further showed that Gαi3 translocation to nuclei, which was important for DNA damage repair. These results together imply that Gαi3 over-expression in human glioma cells could be a key irradiation-resistance factor.

Irradiation-induced DNA damage will initiate DNA repair pathway [20–22]. There are at least two major signaling pathways that could possibly repair DNA damages, including the non-homologous end joining (NHEJ) pathway and the homologous recombination (HR) pathway [20–22]. In the process of NHEJ, Ku70/80 proteins will sense and bind to ends of the DNA termini in a structure-specific manner, which is followed by the recruitment and activation of DNA-PKcs [28, 29]. Afterwards, DNA ligase IV-XRCC4 complex is recruited to repair damaged DNA [28–30]. HR pathway is the second major pathway for DNA DSB repair [20–22]. After DNA damage, the Mre11/Rad50/Nbs1 (MRN) complex is recruited to the DNA ends, which then activates ATM and other DNA damage response proteins to repair broken DNA [30].

It is known that DNA-PKcs is vital in the repair of damaged DNA by irradiation [21, 22, 24, 31, 23, 24]. DNA-PKcs is a phosphatidylinositol-3-kinase (PI3K)-like protein kinase (PIKK) family kinase protein, which is activated following irradiation-induced DNA double-strand breaks (DSBs) [23, 24]. DNA-PKcs silence, depletion or mutation will disrupt DNA repair mechanism, causing irradiation-sensitization [21, 22, 24, 31]. On the other hand, over-expression and/or constitutive activation of DNA-PKcs could inhibit irradiation-induced DNA damage repair, leading to irradiation resistance [20, 30]. Indeed, DNA-PKcs expression is often elevated in glioma [32, 33] and other malignancies [34], and its upregulation in malignancy often correlates with irradiation resistance. Further studies suggest that DNA-PKcs expression level could be serve as a predictor for irradiation sensitivity in human cancer [35].

In the current study, we discovered an unique function of Gαi3: Irradiation in A172 cells induced Gαi3 translocation to nuclei, where it formed a complex with local protein DNA-PKcs. The complexation between Gαi3 and DNA-PKcs was apparently crucial for DNA repair. Gαi3 silence, depletion or dominant-negative mutation significantly potentiated irradiation-induced DNA damages. Reversely, expression of the constitutively-active Gαi3 inhibited DNA damage by irradiation in A172 cells. Future studies will be needed to further explore the detailed mechanisms of Gαi3's function in DNA damage repair.

## MATERIALS AND METHODS

### Reagents

All the antibodies of the current study were described previously [9, 10, 36, 37], and were provided by the Cell Signaling Tech (Shanghai, China) and Santa Cruz Biotech (Shanghai, China). The reagents for cell culture were purchased from Gibco (Shanghai, China). Puromycin was obtained from Sigma (Shanghai, China).

### Cell lines

Wild-type (WT) and Gαi3 knockout (KO) mouse embryonic fibroblasts (MEFs) were described previously [9–11]. Human glioma A172 cell line was purchased from the Cell Bank of Fudan University (Shanghai, China). Cells were cultured in routine DMEM medium, with 10% fetal bovine serum (FBS) in the CO_2_ incubator.

### Irradiation

Cells were irradiated with a 137Cs gamma rays source at a dose rate of 1.25 Gy/min (MDS Nordion Gammacell Irradiator).

### Western blotting analysis

Following the applied treatment, cells were lysed using the lysis buffer described [9, 10, 36]. Aliquots of 30 μg of protein per treatment were separated by 7.5–10% SDS-PAGE gels, and were transferred to the PVDF membrane (Millipore, Bedford, MA). The membrane was then incubated with indicated primary antibody and corresponding second antibody. Antibody-antigen binding was detected by the ECL reagents (Amersham Biosciences). Each band was quantified through Image J software (NIH). Isolate of nuclei-localized proteins was described previously [15, 16]. For all the Western blotting assay, each lane was loaded with exact same amount of quantified protein lysates (30 μg per sample). Same set of lysate samples were run in sister gels to test different proteins.

### Co-immunoprecipitation (Co-IP)

The detailed protocol was described in our previous studies [9, 36]. Briefly, aliquots of 500 μg of nuclei-localized protein lysates from each treatment were pre-cleared with protein A/G beads (30 μL per sample, Sigma). The pre-cleared lysate samples were then incubated with anti-DNA-PKcs antibody [38] overnight. Protein A/G beads (Sigma) were then added again, and the lysates were incubated for 2 hours at 4°C. The beads were washed, and DNA-PKcs-Gαi3 association was then detected by Western blotting assay.

### Gαi3 shRNA

The two lentiviral Gαi3 shRNAs (“−a/−b”) were again purchased from Genechem (Shanghai, China), with the targeted sequences 5′-TCAATCATTCTCTTCCTTA-3′ (Gαi3 shRNA-a) and 5′-CCTCAGTGATTATGACCTT-3′ (Gαi3 shRNA-b), respectively. The lentiviral shRNA was added directed to the cells for 24 hours, puromycin (0. 5 μg/mL, 8 days) was added to select the stable cells. Gαi3 knockdown was confirmed by the Western blotting assay. Same amount of lentiviral scramble shRNA (“scr-shRNA”, Santa Cruz, sc-108080) was added to the control cells.

### Gαi3 over-expression or mutation

The wild-type Gαi3 (-Flag), the constitutively-active-Gαi3 (CA-Gαi3-GFP-puro, Q204L), the dominant-negative Gαi3 (DN-Gαi3-GFP-puro, G202T), and the empty vector (pGCL-GFP-puro, GeneChem) were described previously [9–11]. The construct was transfected to A172 cells by Lipofectamine 2000 reagents [10]. After 24 hours, cells were subjected to puromycin (0.5 μg/mL, 8 days) selection. Expression of the target protein (Gαi3) in stable cells was always tested by Western blotting assay.

### Cell growth, survival and apoptosis assay

MTT assay of cell growth, clonogenicity assay of cell growth, and trypan blue staining of cell death, as well as Histone DNA apoptosis ELISA assay, Annexin V FACS assay of cell apoptosis and TUNEL nuclei staining assay of cell apoptosis were described in detail in our previous studies [9, 10, 16, 17, 36, 37, 39, 40, 41].

### γ-H2AX FACS assay of cellular DNA damage

After irradiation, cells were trypsinized and fixed in ice-cold ethanol. Afterwards, cells were incubated with a mouse monoclonal anti-γ-H2AX antibody (Cellular Signaling Tech, Shanghai, China) for 12 hours, and then incubated with a FITC-conjugated anti-mouse secondary antibody (Cell Signaling Tech). Cells were then subjected to FACS assay to determineγ-H2AX percentage, which indicates DNA damage intensity [27].

### Statistical analysis

The data were presented as means ± standard deviation (SD) of one whole set of experiment. All experiments were repeated at least three times, with similar results obtained in each repeat. Statistical differences were analyzed by one-way ANOVA and multiple comparisons with the post hoc Bonferroni test (SPSS version 18.0). Values of *p* < 0.05 were considered as statistically significant.

## CONCLUSIONS

In summary, these results indicate a pivotal function of Gαi3 in irradiation-resistance in human glioma cells. Gαi3 could be a novel oncotarget for irradiation sensitization for glioma.
